# *In vitro* Infectivity of Strains Isolated From Dogs Naturally Infected With *Leishmania infantum* Present a Distinct Pathogenic Profile in Hamsters

**DOI:** 10.3389/fmed.2020.00496

**Published:** 2020-08-28

**Authors:** Lucilene Aparecida Resende, Rodrigo Dian de Oliveira Aguiar-Soares, Nádia das Dores Moreira, Sidney de Almeida Ferreira, Mariana Ferreira Lanna, Jamille Mirelle de Oliveira Cardoso, Fernando Augusto Siqueira Mathias, Wendel Coura-Vital, Reysla Maria da Silveira Mariano, Jaqueline Costa Leite, Patricia Silveira, Tatiane Furtado de Carvalho, Renato Lima Santos, Denise da Silveira-Lemos, Olindo Assis Martins-Filho, Walderez Ornelas Dutra, Alexandre Barbosa Reis, Rodolfo Cordeiro Giunchetti

**Affiliations:** ^1^Laboratório de Biologia das Interações Celulares, Departamento de Morfologia, Instituto de Ciências Biológicas, Universidade Federal de Minas Gerais, Belo Horizonte, Brazil; ^2^Laboratório de Imunopatologia, Núcleo de Pesquisas em Ciências Biológicas, Universidade Federal de Ouro Preto, Ouro Preto, Brazil; ^3^Departamento de Análises Clínicas, Escola de Farmácia, Universidade Federal de Ouro Preto, Ouro Preto, Brazil; ^4^Programa de Pós Graduação em Ciências Farmacêuticas (Cipharma), Escola de Farmácia, Universidade Federal de Ouro Preto, Ouro Preto, Brazil; ^5^Laboratório de Biologia Parasitária, Faculdade de Ciências da Saúde, Universidade Federal de Lavras, Lavras, Brazil; ^6^Laboratório de Pesquisa em Epidemiologia e Citologia, Departamento de Análises Clínicas, Escola de Farmácia, Universidade Federal de Ouro Preto, Ouro Preto, Brazil; ^7^Departamento de Clínica e Cirurgia Veterinárias, Escola de Veterinária, Universidade Federal de Minas Gerais, Belo Horizonte, Brazil; ^8^Grupo Integrado de Pesquisas em Biomarcadores, Instituto René Rachou, FIOCRUZ, Belo Horizonte, Brazil; ^9^Departamento de Medicina, Universidade José Do Rosário Vellano, UNIFENAS, Belo Horizonte, Brazil

**Keywords:** *Leishmania infantum*, visceral leishmaniasis, experimental infection, pathogenicity, infectivity

## Abstract

Visceral leishmaniasis (VL) is a severe disease caused by *Leishmania infantum*. Dogs are the parasite's main reservoir, favoring its transmission in the urban environment. The analysis of *L. infantum* from infected dogs contributes to the identification of more virulent parasites, thereby supporting basic and applied studies such as vaccinal and therapeutic strategies. We proposed the *in vitro* and *in vivo* characterization of *L. infantum* strains from naturally infected dogs from a VL endemic area based on an infectivity and pathogenicity analysis. DH82 canine macrophages were infected *in vitro* with different strains for infectivity analysis, showing distinct infectivity profiles. The strains that showed greater and lesser infectivity using *in vitro* analyses (616 and 614, respectively) were used to infect hamsters for pathogenicity analysis. The group infected with strain 616 showed 100% survival while the group infected with strain 614 showed 50% after seven months of follow up. Furthermore, the 614 strain induced more noticeable clinicopathological changes and biochemical abnormalities in liver function, along with high inflammation and parasite load in the liver and spleen. We confirmed high variability of infectivity and pathogenicity in *L. infantum* strains from infected dogs. The results support the belief that screening for *L. infantum* infectivity using *in vitro* experiments is inadequate when it comes to selecting the most pathogenic strain.

## Introduction

*Leishmania infantum* (syn. *Leishmania chagasi*) is the etiological agent of visceral leishmaniasis (VL) in Latin America, a severe chronic systemic disease (1–3). More than 90% of VL cases reported in the New World occur in Brazil. Initially, VL presented a rural epidemiological profile, but it has expanded into urban areas in the past few decades (4, 5).

Dogs are the main domestic reservoir for the parasite in various geographical locations, playing an important role in its transmission to humans (6, 7). Moreover, euthanasia has been shown to have limited effects in controlling visceral leishmaniasis in Brazil (8).

A major problem in controlling the transmission of visceral leishmaniasis in endemic areas is that many dogs are asymptomatic, even when presenting high levels of skin parasitism (9, 10). Thus, asymptomatic dogs are highly competent transmitters of *L. infantum*, making canine visceral leishmaniasis (CVL) more important epidemiologically than human VL (9, 11, 12).

Manifestation of the disease depends on the virulence of the *Leishmania* strain, as well on the genetic background and immune status of the host (13–16). A study conducted by Cunha et al. (17) characterized the biology and infectivity of viscerotropic and dermatotropic strains of *L. infantum* isolated from HIV+ and HIV- patients in the VL murine model, and demonstrated that *in vivo* and *in vitro* virulence are intrinsic characteristics of each strain. Another study that evaluated the *ex vivo* virulence of the *L. infantum* isolated from *Phlebotomus perniciosus* captured in an endemic area of Madrid, Spain, demonstrated that *L. infantum* strains exhibited high virulence, and were associated with cytokine production and enzymatic activities involved in the VL pathogenesis (18). Moreover, it has been shown that *in vitro* infection of human monocytes by *Leishmania* strains induced distinct immunological characteristics and the host cell's ability to control *Leishmania* (19). Based on these data, it is reasonable to hypothesize that parasite transmission in endemic areas could be related to distinct infectivity and virulence profiles.

In this study, we examined eight different *L. infantum* strains from dogs with CVL, characterizing them according to *in vitro* (*L. infantum* infectivity in DH82 cell linage) and *in vivo* (pathogenicity evaluation in *Mesocrisetus auratus*- hamster model) approaches. Our results clearly show that strains isolated from naturally infected dogs display distinct *in vitro* infectivity as well as pathogenic profiles in hamsters.

## Materials and Methods

### Ethical Approval

The protocols for experimental *L. infantum*-infection in hamsters were approved by the Ethical Committee on Animal Research of the Universidade Federal de Ouro Preto, State of Minas Gerais, Brazil (approval ID number 2012/37).

### *L. infantum* Parasites Used for *in vitro* and *in vivo* Analysis

Eight *L. infantum* strains were used in *in vitro* evaluations for this study: PP75 (WHO/MHOM/BR/74/PP75) and seven wild-type strains isolated from the bone marrow of naturally *L. infantum*-infected dogs from Belo Horizonte, Minas Gerais, as described in Table 1. All parasites used in this study were confirmed to be *L. infantum* by PCR-RFLP according to Coura-Vital et al. (20).

**Table 1:** Clinical features of canine visceral leishmaniasis after isolation of *L. infantum* strains from naturally *L. infantum*-infected dogs.

**Wild strains**	**Clinical signs observed in dogs during *L. infantum* isolation procedures**
571	Moderate weight loss
591	Enlargement of popliteal lymph nodes, seborrheic dermatitis, ulceration in the left paw, jaundice, splenomegaly
592	Intense weight loss, seborrheic dermatitis, periocular dermatitis, onychogryphosis, enlargement of pre-scapular lymph nodes
593	Intense weight loss, seborrheic dermatitis, dermatitis in the periocular, ears, ribs and tail regions, onychogryphosis, enlargement of pre-scapular lymph nodes
610	Moderate weight loss, seborrheic dermatitis, dermatitis in the periocular, ears, ribs and tail regions, onychogryphosis, enlargement of all lymph nodes
614	Seborrheic dermatitis, dermatitis in the periocular, ears (with bleeding at the tip) and tail regions, localized ulceration, enlargement of all lymph nodes
616	Moderate weight loss, dermatitis in the ears, onychogryphosis, enlargement of all lymph nodes

Parasites were grown in LIT and NNN (McNeal, Novy & Nicolle) medium up to eight *in vitro* passages to preserve the most important parasite characteristics regarding infectivity and pathogenicity profiles, in addition to providing appropriate adaptation in the media culture. Stationary growth phase promastigotes, comprising predominantly metacyclic promastigotes (21–23), were obtained from an initial inoculum of 10 mL of LIT medium containing between 10^7^ and 10^8^ promastigotes/mL in logarithmic growth. The promastigotes were added to 40 mL of NNN/LIT culture medium and stored at 23 ± 1°C.

### *In vitro* Experimental Infection of Canine Macrophages

The DF82 canine macrophage cell line (DS Pharma Biomedical, Osaka, Japan) was placed over circular coverslips (15 mm; Glasscyto, Brazil) on 24-well plates for 3 h. Promastigotes, in stationary phase, were added at 4:1 and 10:1 parasite:host cell ratios. The cultures were incubated for 3, 24, and 48 h at 37°C in 5% CO_2_. The amastigotes were counted in 300 macrophages using optical microscopy at 1,000X magnification. CFSE (Carboxyfluorescein succinimidyl diacetate ester; *Vybrant*® CFDA-SE *Cell tracer kit*, Invitrogen) was used to label parasites at a concentration of 2.8 g/mL (24). After washing twice with 15 mL RPMI, the pellet was suspended in 1 mL of RPMI 1640 and added in 4:1 and 10:1 parasite:host cell ratios. Labeled *Leishmania* were incubated together with macrophages in CO_2_ at 37°C for 3 and 24 h in polystyrene tubes (Becton Dickinson, Franklin Lakes, USA) to grow as non-adherent cells. Samples were centrifuged and ressuspended in 200 μL of fix solution, MaxFacsFix (10.0 g/L paraformaldehyde, 10.2 g/L sodium cacodylate, and 6.65 g/L sodium chloride, pH 7.2). A FACScan flow cytometer (Becton Dickinson, San Diego, USA) was used for acquisition, and data were analyzed using FlowJo® (FlowJo, LLC, Oregon, USA).

The *in vitro* experiments were used to select the most and least infective strains for use in a pathogenicity analysis in hamsters. The criteria used to select the parasites were based on: (i) the percentage of infection (low or high) consistently observed in optical microscopy and flow cytometry method, regardless of the difference in the intrinsic magnitude of data generated by microscopy and flow cytometry and (ii) isolates with intermediate infectivity patterns that were considered difficult to be categorized as most or least infective and, thus, not segregated into these categories.

### *In vivo* Experimental Infection in Hamsters

Hamsters were experimentally infected with the wild strains chosen for presenting the lowest and highest infectivity profile after *in vitro* analysis, plus a reference strain (PP75). One-month-old male Syrian golden hamsters (*Mesocricetus auratus*), weighing ~50–80 g, were obtained from the Animal Science Center at the Universidade Federal de Ouro Preto (UFOP). Experimental infection was done at the end of seven days of promastigote cultivation [stationary phase presenting predominantly metacyclic forms as described by (23)] that were transferred to sterile polypropylene tubes (FalconH, Becton Dickinson, USA) and centrifuged at 900x*g* for 15 min. The parasites were adjusted to 10^7^ promastigotes per inoculum. The experimental infection was made by intracardiac (i.c.) route, with the following strains (*n* = 10/group): PP75 (WHO/MHOM/BR/74/PP75), 614, and 616 wild-type strains. Uninfected animals were used as a control group (C). Euthanasia was carried out 210 days after infection by injection, following anesthesia with intraperitoneal barbiturate (Thiopental® at 90 mg/kg of body weight). Hamsters were clinically monitored throughout the experiment for signs of suffering due to *Leishmania* infection.

### Hematological and Biochemical Parameters in Hamster Model

The hematological and biochemical parameters were analyzed as previously described (25). The samples were analyzed using the Auto Hematology Analyzer apparatus (Mindray BC-2800, Hamburg, Germany) for an overall analysis of leukocytes, erythrocytes, hematocrit, and hemoglobin. A differential cell count was performed on blood smears stained with Panotic Quick InstantProv (Newprov®) and evaluated by optical microscopy at 1,000X magnification. Biochemical analysis was based on evaluation of urea and creatinine levels for kidney function. Moreover, measures of the enzymes AST (aspartate aminotransferase) and ALT (alanine aminotransferase) were obtained to assess hepatic function. Biochemical analyses were performed using the Biochemical System Auto (CELM SBA-200, Barueri, SP, Brazil) and commercials Labtest Kits (Labtest Diagnostica SA, Lagoa Santa, MG, Brazil), following the manufacturer's instructions.

### Histological Evaluation

The size and appearance of the spleen and liver were macroscopically recorded during necropsy to check rounded edge and/or congestion resulting from organ enlargement and described as splenomegaly and hepatomegaly, respectively. Liver and spleen samples were fixed in 10% buffered formalin for 24 h, paraffin embedded, and 4 μm thick sections stained with hematoxylin and eosin (HE). Inflammatory, degenerative, and hyperplastic lesions were classified according to the following scores: 0 (absent), 1 (mild), 2 (moderate), and 3 (severe).

### Immunohistochemistry

Immunohistochemistry was performed as previously described to evaluate parasite load (23) in sections of the spleen and liver (26). The numbers of immunolabeled *Leishmania* amastigotes in the spleen and liver sections were classified according to the following scores: 0 (absent), 1 (mild, 1–50 amastigotes per microscopic field under 400x magnification), 2 (moderate, 51–100 per microscopic field under 400x magnification), and 3 (severe, more than 101 amastigotes per microscopic field under 400x magnification).

### DNA Extraction and Quantitative Real-Time PCR

DNA extraction and quantitative real time PCR were performed as previously described (21), using 20 mg of tissue (spleen or liver). The concentration and purity of DNA was determined using spectrophotometer (NanoVue Plus, GE Healthcare Products, Piscataway, NJ, USA) based on A_260_/A_280_ and A_260_/A_230_ measures. To quantify parasite burdens, primers were used as described by Bretagne et al. (27), which amplified a 90-bp fragment of a single-copy of DNA polymerase gene of *L. infantum* (GenBank accession number AF009147). Standard curves were prepared for each run using known quantities of promastigotes (PP75 strain) seeded in cultures. Purified DNA was diluted from 10^6^ until 1.0 parasite/μL following 1:10 ratio. Each curve point was performed in triplicate. The quantification cycles (Cq) indicated the quantities of parasites per 20 ng of total extracted DNA by interpolation of the standard curve. Afterwards, the total number of parasites was calculated for the total quantity of extracted DNA and divided by 20 mg of tissue used in DNA purification. The final result was estimated as the number of amastigotes/mg of tissue. Reactions were processed and analyzed in an ABI Prism 7500-Sequence Detection System (Applied Biosystems, USA).

### Statistical Analysis

Statistical analyses were conducted using Prism 5.0 software package (Prism Software, Irvine, CA, USA). Data normality was assessed using the Kolmogorov-Smirnoff test. One-way analysis of variance (ANOVA) and Tukey post-test were used to investigate differences between groups. Considering the nonparametric nature of real time PCR data, the Kruskal-Wallis test was used, followed by Dunn's test. Comparisons that returned a *P*-value < 0.05 were considered statistically significant.

## Results

### The 616 Strain Presented a Highlighted Pattern of *in vitro* Infectivity

**Figure 1:**
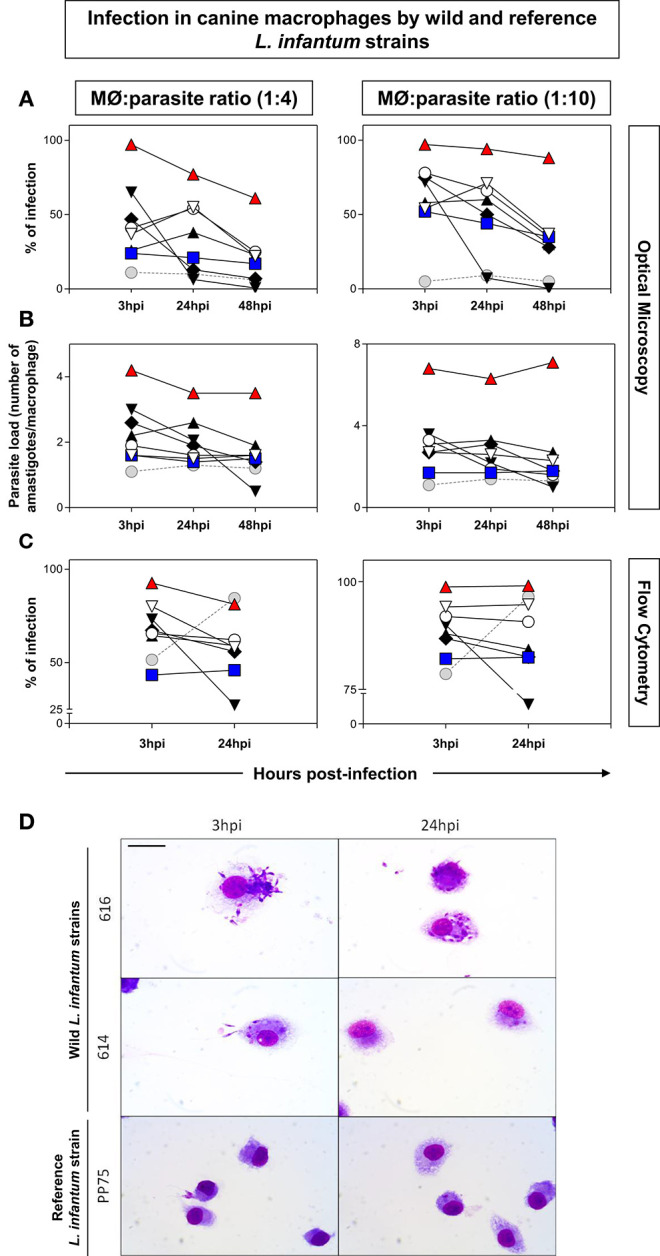
Measure of infection in canine macrophages by wild [571 (
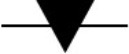
), 591 (
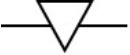
), 592 (
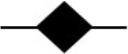
), 593 (
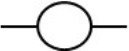
), 614 (
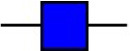
), 610 (
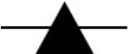
), (616 (
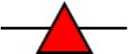
)], and reference [(PP75, 
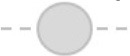
; MHOM/BR/74/PP75)] *L. infantum* strains. The 1:4 (left panel) and 1:10 (right panel) ratios correspond to the proportions between DH82 macrophages (MØ) and parasites. Distinct proportions of macrophage: promastigotes were displayed in the left (1:4) and right panels (1:10). The y-axis shows the percentage of infection **(A,C)** and parasite load **(B)**. The x-axis shows the time periods (3, 24, and 48 h) post-infection. The data were obtained by optical microscopy **(A,B)** or flow cytometry using parasites labeled with CFSE **(C)**. The data were displayed as descriptive analyses given they are representative of each strain. **(D)**: Immortalized canine DH82 macrophages infected with wild (614 and 616) and reference (PP75) L. infantum strains. The macrophages were observed 3 and 24 h post-infection. Bar = 20 μm.

It was observed that the 616 strain showed the highest percentage of infection and increased parasite load (Figures 1A–C). The analysis employing parasites labeled with CFSE was performed 3 and 24 hpi using the 1:4–1:10 parasite:macrophage ratios. The results confirmed that the 616 strain was the most infective, while the 614 strain was the least infective (Figures 1A–C). Figure 1D illustrates the interaction of parasites with macrophages by optical microscopy, showing the polarized profile as high and low infectivity in the 616 and 614 strains, respectively.

### Unlike the *in vitro* Experiments, the 614 Strain Displayed High Pathogenicity in Infected Hamsters Inducing 50% of the Deaths After 7 Months of Infection

**Figure 2:**
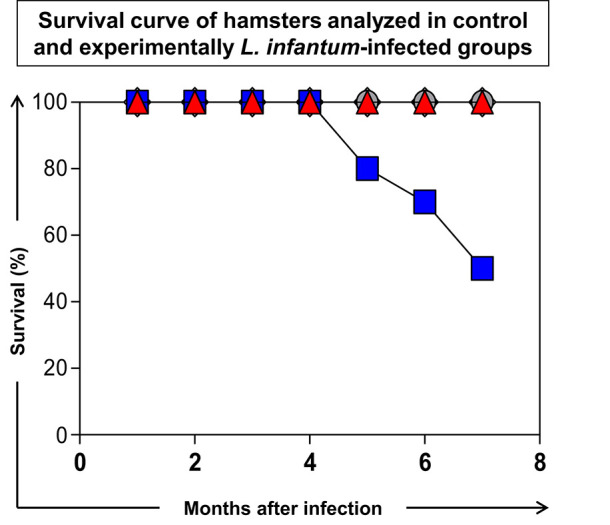
Survival curve of hamsters analyzed in control (C: 
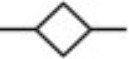
) group and experimentally *L. infantum*-infected groups (wild strains: 614, 
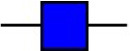
 and 616, 
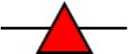
; reference strain: PP75, 
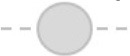
). The data were displayed as descriptive analyses given they are representative of each strain. All the groups were analyzed during 210 days after infection and euthanasia was performed for necropsy evaluation.

**Figure 3:**
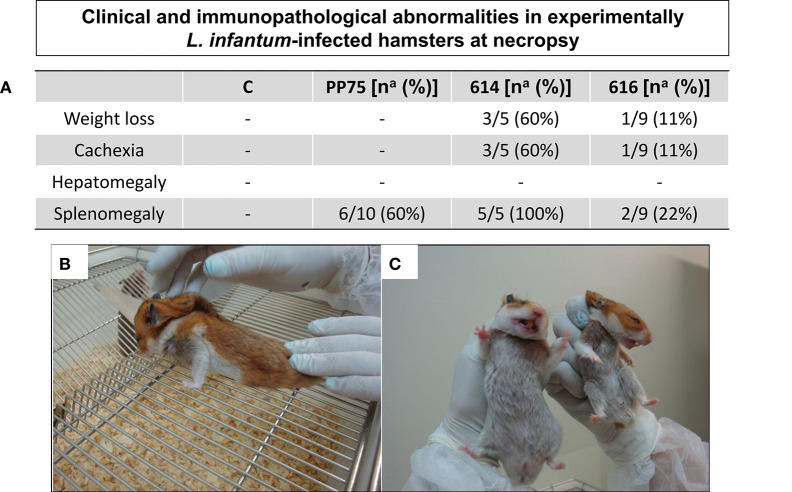
Clinical/immunopathological abnormalities observed in experimentally *L. infantum*-infected hamsters at necropsy. **(A)** Frequency [number of positive hamsters in relation to total analyzed animals – n^a^; and respective percentage (%)] of immunopathological changes according the analyzed groups [C, control; experimentally *L. infantum*-infected wild (614 and 616) and reference (PP75) strains]. Animals from 614 (*n* = 5) group died before the end of the experiment, as demonstrated in the survival rate displayed in Figure 2; **(B)** Hamster infected with 614 strain displaying weight loss and cachexia; **(C)** normal hamster (at left) and an animal with cachexia and weight loss (at right).

Seven months after experimental infection, the uninfected animals (C) and animals infected with the 616 strain showed 100% survival rate. The animals infected with 614 strains showed a 50% survival rate at seven months postinfection (Figure 2), and exhibited more severe clinical signs (Figure 3). Moreover, 60% of the animals infected with 614 strain had low weight and cachexia at the time of necropsy. In the group infected with 616 strain, 11 showed these signs (Figure 3A). The cachectic appearance in which these animals were found is illustrated in Figures 3B,C. All hamsters infected with 614 strain and 22% of 616 strain showed splenomegaly at the time of necropsy (Figure 3A).

### Hamsters Infected With the 614 Strain Exhibited Severe Anemia and Lymphopenia

Infection with the 614 strain induced severe anemia as observed by reduced levels (*P* < 0.05) of erythrocytes, hemoglobin, and hematocrit, as compared to the other experimental groups (C, PP75, and 616) (Table 2). Moreover, infection with the 614 strain produced lower (*P* < 0.05) lymphocytes counts in comparison to the PP75 group (Table 2).

**Table 2:** Hematological profile from non-infected animals (C; control group) and experimentally *L. infantum-*infected groups (reference strain: PP75; and wild strains: 614 and 616).

**Hematological Parameters**	**Groups**			
	**C**	**PP75**	**614**	**616**
Neutrophils (cells/μL)	680.3 ± 397.1	951.6 ± 582.3	256.5 ± 144.4	812.2 ± 585.5
Eosinophils (cells/μL)	39.7 ± 25.4	67.0 ± 46.2	31.2 ± 29.0	50.4 ± 38.1
Lymphocytes (cells/μL)	2,012 ± 659.4	2,426 ± 540.4	1,568 ± 1,011^b^	2,142 ± 796.3
Monocytes (cells/μL)	61.7 ± 43.6	65.4 ± 35.6	32.0 ± 29.3	65.6 ± 76.6
Erythrocytes (10^6^/μL)	6.4 ± 1.1	7.2 ± 0.1	3.9 ± 2.1^a^^,^ ^b^^,^ ^d^	6.6 ± 0.8
Hemoglobin (g/dL)	13.1 ± 1.4	14.0 ± 0.5	7.9 ± 4.3^a^^,^ ^b^^,^ ^d^	13.1 ± 1.7
Hematocrit (10^6^/μL)	27.7 ± 2.9	29.4 ± 1.5	17.1 ± 8.6^a^^,^ ^b^^,^ ^d^	27.4 ± 3.6
VCM (fL)	40.9 ± 1.1	40.8 ± 1.0	35.1 ± 14.7^a^^,^ ^b^	41.3 ± 1.0
HCM (pg)	19.4 ± 0.6	19.5 ± 0.5	16.1 ± 6.7	19.8 ± 0.4

*The measurements exhibited in the control group were used as reference values. Statistical differences were displayed as “a,” “b,” and “d” for the “C,” “PP75,” “616” groups, respectively. All data are shown as mean ± standard deviation*.

### The 614 Strain Induced an Increase in the Serum AST and ALT Levels, a Clinical Marker of Hepatic Dysfunction Regarding Severe Visceral Leishmaniasis

Biochemical evaluations consist of liver function tests, including the measurement of AST and ALT, and renal function tests based on urea and creatinine levels. Notably, animals infected with the 614 strain presented an increase (*P* < 0.05) in AST and ALT serum levels in relation to the C and PP75 groups (Table 3). Moreover, the 616 strains induced increased ALT levels in comparison to the PP75 strain (Table 3).

**Table 3:** Biochemical profile from non-infected animals (C; control group) and experimentally *L. infantum-*infected groups (reference strain: PP75; and wild strains: 614 and 616).

**Biochemical Parameters**	**Groups**			
	**C**	**PP75**	**614**	**616**
AST	87.3 ± 73.0	83.8 ± 30.1	245.6 ± 112.1^a^^,^ ^b^	163.6 ± 117.6
ALT	97.1 ± 39.1	78.4 ± 19.2	170.8 ± 125.9	134.1 ± 50.1
Ureia	47.6 ± 3.8	39.2 ± 2.5	94.3 ± 96.8	50.8 ± 10.7
Creatinine	0.4 ± 0.0	0.3 ± 0.05	0.3 ± 0.1	0.3 ± 0.05

*The measurements exhibited in the control group were used as reference values. Statistical differences were displayed as “a” and “b,” for the “C” and “PP75” groups, respectively. All data are shown as mean ± standard deviation*.

### Infection Triggered by the 614 Strain Showed the Worst Histopathological Findings in Hepatic and Splenic Compartments

**Figure 4:**
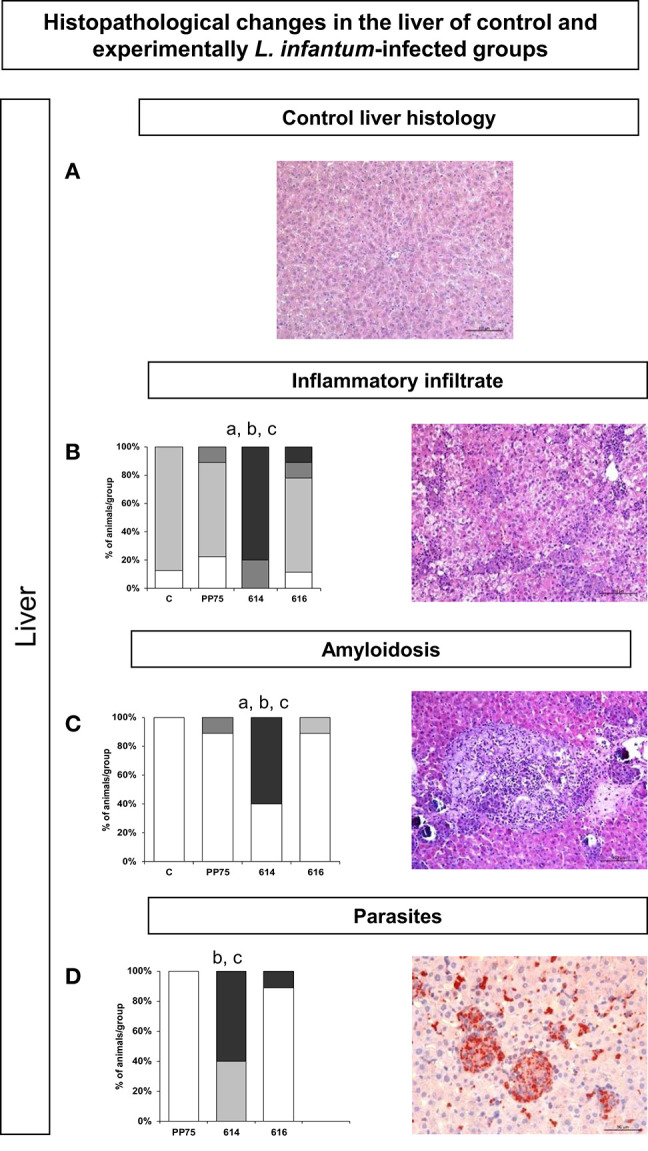
Frequency and histopathological changes in the liver of control group and experimentally *L. infantum*-infected groups (reference strain: PP75; and wild strains: 614 and 616). The intensity of histopathological changes was graded as absent (−, 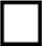), mild (+, 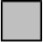), moderate (++, 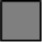), and severe (+++, 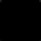). The histopathology analysis was performed for inflammatory infiltrate **(B)**, amyloidosis **(C)**, and parasitism **(D)**. Images obtained from optical microscopy illustrate each analysis of HE-stained **(A–C)** and immunohistochemistry **(D)** slides for all evaluated histopathological parameters. Representative images **(B–D)** correspond to animal samples from the group most affected by the infection (614). An image of the control liver histology **(A)** was included to illustrate the normal histological condition in hamsters. Statistically significant differences are highlighted by the letters “a,” “b,” and “c” as compared to Control *(C)*, PP75, and 616, respectively, at *P* < 0.05.

In hamsters infected with the 614 strain, the intensity of inflammatory infiltrate (Figure 4B) in the liver, consisting of lymphocytes, hstiocytes, plasma cells, randomly distributed neutrophils, amyloidosis (Figure 4C), and parasitism (Figure 4D) identified by immunohistochemistry were significantly higher as compared to the other groups (C, PP75, and 616). Figure 4A represents the control group.

**Figure 5:**
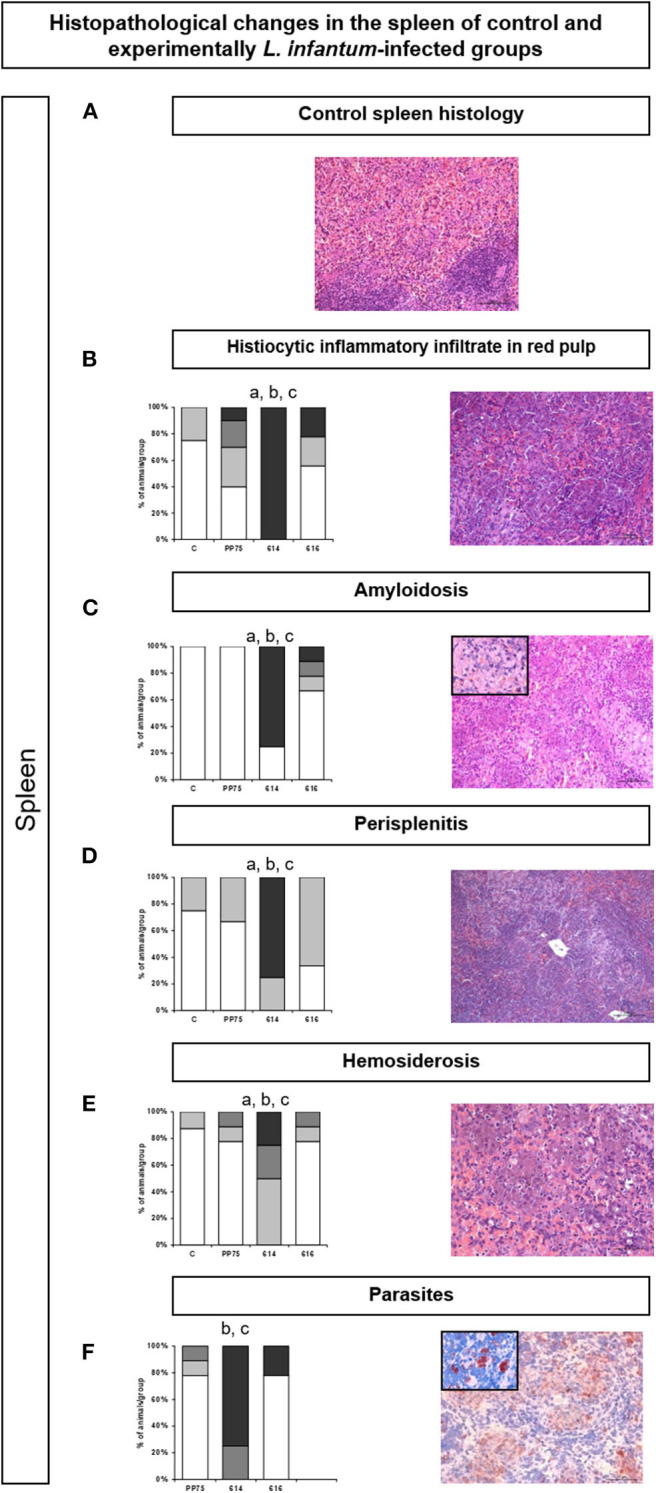
Frequency and histopathological changes in the spleen of control group and experimentally *L. infantum-*infected groups (reference strain: PP75; and wild strains: 614 and 616). The intensity of histopathological changes was graded as absent (–, 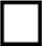), mild (+, 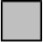), moderate (++, 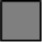), and severe (+++, 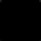). The histopathology analysis was performed for histiocytic inflammatory infiltrate in the red pulp **(B)**, amyloidosis **(C)**, perisplenitis **(D)**, hemosiderosis €, and parasitism **(F)**. Images obtained from optical microscopy illustrate each analysis of HE-stained **(A–E)** and immunohistochemistry **(F)** slides for all evaluated histopathological parameters. Representative images **(B–F)** correspond to animal samples from the group most affected by the infection (614). An image of the control spleen histology **(A)** was included to illustrate the normal histological condition in hamsters. Statistically significant differences are highlighted by the letters “a,” “b,” and “c” as compared to Control (C), PP75, and 616, respectively, at *P* < 0.05.

Similarly, in the spleen, the 614 strain induced significantly more intense histopathological changes as illustrated by high (*P* < 0.05) histiocytic infiltrate in the red pulp (Figure 5B), amyloidosis (Figure 5C), perisplenitis (Figure 5D), hemosiderosis (Figure 5E), and numbers of parasites through immunohistochemistry (Figure 5F). Figure 4A represents the control group.

### The 614 Strain Was Able to Infect All Hamsters According to an Analysis of the Liver and Spleen, Plus a High Parasite Load in the Liver by qPCR Analysis

**Figure 6:**
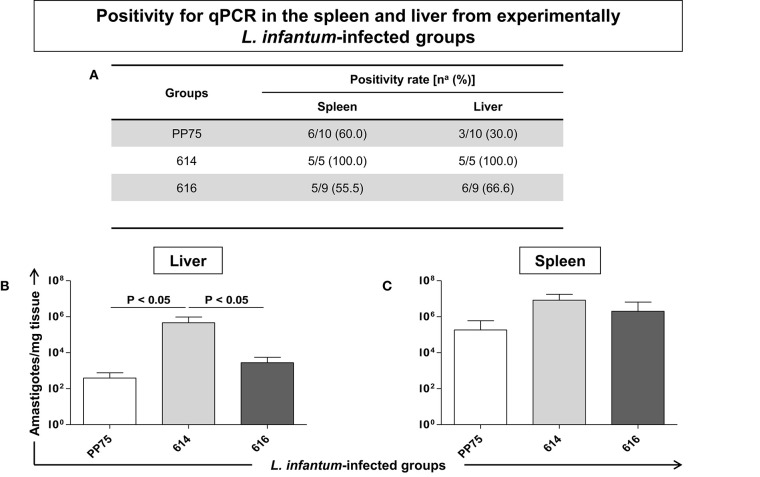
Positivity for qPCR in the spleen and liver **(A)** from experimentally *L. infantum*-infected groups (reference strain: PP75; and wild strains: 614 and 616). **(A)** Number of positive hamsters in relation to total analyzed animals. Animals from 614 (*n* = 5) group died before the end of the experiment, as demonstrated in the survival rate displayed in Figure 2. The y-axis shows the number of amastigotes per milligram of tissue in the liver **(B)** and spleen **(C)**. The x-axis shows the *L. infantum*-infected groups (reference strain: PP75 and wild strains: 614 and 616). The significant difference (*P* < 0.05) is indicated on the graph by connecting lines.

The group infected by the 614 strain presented 100% positivity in the spleen and liver compartments (Figure 6A). Moreover, the same group showed a high parasite load (*P* < 0.05) in the liver (Figure 6B) as compared to the PP75 and 616 strains. No significant changes were found in the spleen (Figure 6C).

## Discussion

Dogs are the main domestic reservoir of *L. infantum* favoring its transmission in urban areas (6, 7). In this study, we examined different *L. infantum* strains isolated from dogs living in a VL endemic area in Brazil, using *in vitro* and *in vivo* approaches to determine the infectivity and pathogenicity profiles. The wild strains used to infect canine macrophages (DH82) presented distinct virulence profiles. After *in vitro* infection, we selected the two strains with more and less infectivity profiles to perform the pathogenicity experiments in the hamster model. Importantly, we included a reference *L. infantum* strains (MHOM/BR/74/PP75) in all *in vitro* and *in vivo* experiments. Similarly, previous studies have used *L. infantum* PP75 strain as a reference strain to analyze experimental infection in hamsters (21, 22).

More recently, research groups have used *in vitro* macrophage infection as an advantageous alternative in infectivity studies (17, 18, 28). Our previous experiments to standardize the *in vitro* conditions of macrophage and parasite co-cultures demonstrated that incubation after 3, 24, and 48 h resulted in consistent macrophage integrity and parasite growth (data not shown). However, it is important to select the best strain to use in both *in vitro* and *in vivo* experiments, considering the possibility of dichotomous virulence profiles in these two systems. Zhang and Matlashewski (29) demonstrated that the expression profile of the virulence-associated genes showed species-specific and stage-specific differences. The present study demonstrated high variability of infectivity and pathogenicity in *L. infantum* strains. Interestingly, the strain with less infectivity in *in vitro* experiments behaved differently in the *in vivo* analysis, leading to high infectivity and pathogenicity. The data suggests that distinct virulence patterns could occur intrinsically in the species-specific and stage-specific conditions, as recently reported for human monocytes infected with distinct *Leishmania* strains and species (19).

In the present experimental model, it was possible to observe that 616 was the most infective strain in the *in vitro* experiments, a pattern that was reversed in the *in vivo* experiments. Conversely, the 614 strain that presented low *in vitro* infectivity resulted in a high pathogenicity in hamsters. This study corroborates the data obtained by Cunha et al. (17), which showed differences between *in vitro* and *in vivo* infectivity in the murine model attributed to the intrinsic characteristics of each strain.

*In vitro* cultivation of *Leishmania* strains leads to wide variation due to laboratory conditions, becoming highly complex when comparing similar experiments. This may occur due to differences in the culture medium (30), number of passages performed, and time of the culture. Therefore, the promastigote forms generated will be differently enriched in the metacyclic forms (27), resulting in distinct infection patterns. Furthermore, the genetics and immune status of the host play an important role in the severity of the disease (31, 32). However, even with these variables, there is no doubt the different strains display distinct *in vitro* infectivity profiles, as observed in the present experiment.

The route, number of parasites, and time after infection contribute to determining the spectrum of clinical manifestation in experimental infection using the hamster model for VL (21, 22). The 614 strain stood out due to its high infectivity and pathogenicity in hamsters, with lymphopenia and anemia that resulted in 50% of deaths after seven months of infection. In fact, the animals of this group that died showed marked weight loss and cachexia, which lasted for a period of ~1 week until death (data not shown). Importantly, we obtained these isolates from naturally infected dogs in an endemic area and observed that the dog from which isolate 614 induced severe VL clinical signs. The dog from which isolate 616 was obtained showed few clinical signs (data not shown). These data support our hypothesis that isolate 614 is more pathogenic.

It is worth emphasizing that *Leishmania*-specific experimental infection in a hamster model displays many clinical pathological aspects related to canine and human VL. A number of hematological and biochemical biomarkers have been analyzed in natural and experimental VL infection as prognosis biomarkers (21, 22, 25, 33, 34). In this study, hamsters infected with the 614 strain exhibited severe anemia with a reduction in red blood cells, hemoglobin, and hematocrit, along with a reduction in lymphocyte levels. Hematological parameters may have relevance in monitoring the clinical status and definition of therapeutic protocols, usually described as normocytic and normochromic anemia, displaying reduced hemoglobin and hematocrit values (35–39). Regarding the leukocytes, immunosuppression occurs with intense leukopenia, monocytopenia, lymphopenia, and eosinopenia (11, 35, 38, 40–44). Moreover, we described that hamsters infected with the 614 strain exhibited an increase in AST and ALT serum levels. Changes in the concentrations of serum aminotransferase enzymes may indicate liver cell injury (45), so these protein levels are important as a clinical VL biomarker.

In fact, the liver is one of the most affected organs in VL infection, in which the resolution of the acute infection was associated with the development of inflammatory interlobular hepatic granulomas around the Küpffer cells (46–48). According to Moreira et al. (21), increased inflammation and the presence of high numbers of granulomas may be related to intense parasitism in hamsters. Similar results were observed in hamsters infected with the 614 strain, with the liver presenting intense inflammation and amyloidosis associated with a high parasite load. Furthermore, it was observed that the 614 group presented intense inflammatory infiltrate in the spleen, amyloidosis, and hemosiderosis, plus a high parasite load. In contrast, the 616 strain that showed high *in vitro* infectivity did not present relevant clinical/pathological and biochemical/hematological findings, resulting in a mild infection with lower parasite load. Histopathology and PCR experiments agree to show mainly a higher pathogenicity caused by the isolate 614. The differences between the parasitism evaluated by microcopy and PCR could be explained by the fact that these were different organ fragments analyzed. It is important to note that these strains were isolated from naturally *L. infantum*-infected dogs in an VL endemic area. Furthermore, the dogs infected with the 614 strain showed severe clinical signs indicative of VL in contrast to that observed in dogs infected with the 616 strain (data not shown). These data corroborate the high pathogenicity hypothesis related to the 614 strain. Moreover, there are studies showing that the increased gene copy numbers due to chromosome amplification may contribute to alterations in gene expression in response to environmental conditions in the host, providing a genetic basis for disease tropism (49). The analysis of *L. infantum* isolated from naturally infected dogs may contribute to identifying more virulent parasites, thus supporting basic and applied studies as vaccinal and therapeutic strategies. Using a genetic analysis of these strains to identify and characterize the genes expressed could contribute to identifying intrinsic parasite factors regarding distinct virulence and pathogenicity profiles.

## Conclusions

This study demonstrated that *L. infantum* strains, obtained from naturally infected dogs, display different infectivity and pathogenicity profiles. We showed that the strain inducing the lowest infectivity profile (infection frequency and parasite load analyzed in canine macrophages) was able to induce the strongest pathogenicity (mortality, anemia, lymphopenia, clinical signs, histological damage in the liver and spleen, and parasite load in liver) in hamsters. It supports that screening of *L. infantum* infectivity using *in vitro* experiments is inadequate when selecting the best pathogenic strain. Therefore, further studies are warranted to better understand the characteristics of these strains, allowing their use as a tool in research related to the prevention and treatment of VL.

## Data Availability Statement

All datasets generated for this study are included in the article/supplementary material.

## Ethics Statement

The animal study was reviewed and approved by Ethical Committee on Animal Research of the Universidade Federal de Ouro Preto, State of Minas Gerais, Brazil (approval ID number 2012/37).

## Author Contributions

LR, RA-S, JC, PS, TC, and RS conceived and designed the study, contributed to the data analysis, and drafted and revised the manuscript. LR, RA-S, NM, SF, ML, JC, and FM performed the experiments. WC-V provided the 614 and 618 strains. LR, DS-L, OM-F, WC-V, WD, AR, and RG wrote and revised the manuscript. All authors contributed to the article and approved the submitted version.

## Conflict of Interest

The authors declare that the research was conducted in the absence of any commercial or financial relationships that could be construed as a potential conflict of interest.
